# Lipids and Lipid-Processing Pathways in Drug Delivery and Therapeutics

**DOI:** 10.3390/ijms21093248

**Published:** 2020-05-04

**Authors:** Milica Markovic, Shimon Ben-Shabat, Aaron Aponick, Ellen M. Zimmermann, Arik Dahan

**Affiliations:** 1 Department of Clinical Pharmacology, School of Pharmacy, Faculty of Health Sciences, Ben-Gurion University of the Negev, Beer-Sheva 8410501, Israel; milica@post.bgu.ac.il (M.M.); sbs@bgu.ac.il (S.B.-S.); 2 Department of Chemistry, University of Florida, Gainesville, FL 32603, USA; aponick@chem.ufl.edu; 3 Department of Medicine, Division of Gastroenterology, University of Florida, Gainesville, FL 32610, USA; Ellen.Zimmermann@medicine.ufl.edu

**Keywords:** lipid, fatty acid, glyceride, steroid, phospholipid, oral drug absorption, prodrug, phospholipase A_2_ (PLA_2_)

## Abstract

The aim of this work is to analyze relevant endogenous lipid processing pathways, in the context of the impact that lipids have on drug absorption, their therapeutic use, and utilization in drug delivery. Lipids may serve as biomarkers of some diseases, but they can also provide endogenous therapeutic effects for certain pathological conditions. Current uses and possible clinical benefits of various lipids (fatty acids, steroids, triglycerides, and phospholipids) in cancer, infectious, inflammatory, and neurodegenerative diseases are presented. Lipids can also be conjugated to a drug molecule, accomplishing numerous potential benefits, one being the improved treatment effect, due to joined influence of the lipid carrier and the drug moiety. In addition, such conjugates have increased lipophilicity relative to the parent drug. This leads to improved drug pharmacokinetics and bioavailability, the ability to join endogenous lipid pathways and achieve drug targeting to the lymphatics, inflamed tissues in certain autoimmune diseases, or enable overcoming different barriers in the body. Altogether, novel mechanisms of the lipid role in diseases are constantly discovered, and new ways to exploit these mechanisms for the optimal drug design that would advance different drug delivery/therapy aspects are continuously emerging.

## 1. Introduction

**Figure 1:**
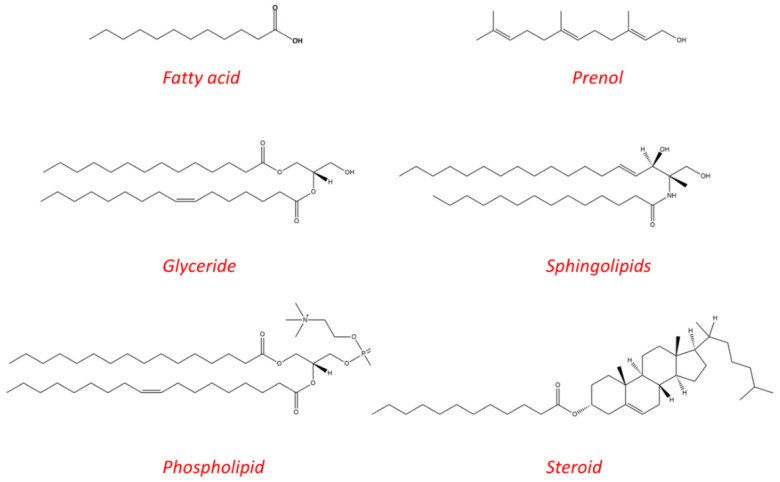
Main lipid categories: examples of chemical structures.

Lipids are hydrophobic biomolecules, which include fatty acids (FA), glycerides, phospholipids (PL), sterols, sphingolipids, and prenol lipids (Figure 1) [1]. Lipids play an important role in energy metabolism and storage, as structural components, in signaling, and as hormones. The disruption of lipid metabolic enzymes and pathways occurs in many disease such as cancer, diabetes, infectious, neurodegenerative, and inflammatory diseases [2]. The aim of this work is to elucidate the effect of lipids, and lipid excipients on drug absorption, to describe the metabolic lipid pathways and to demonstrate the role that lipids have in many pathological conditions, as well as their endogenous pharmacological activity. An additional section is dedicated to lipidic prodrugs that can exploit lipid processing pathways in order to achieve their effect. The presence of dietary lipids or lipids from drug formulations/lipidic prodrugs can influence drug absorption by incorporating to the natural lipid metabolic pathways. Hereinafter, we provide a small overview of the lipid influence on drug absorption.

Molecular revolution, such as development of in-vitro high-throughput screening methods and combinatorial chemistry, resulted in a high number of poor aqueous solubility molecules to be selected as drug candidates. Nearly 40% of all novel drug candidates are lipophilic and demonstrate low water solubility [3,4]. Following oral administration, drugs encounter various obstacles on their way to the blood circulation. Absorption is a process, in which orally administered compounds travel from the gastrointestinal (GI) lumen into the intestinal membrane and enter systemic circulation/become bioavailable. Following ingestion and prior to permeation, the drug has to be dissolved in the GI milieu and turn into a molecular form close to the intestinal membrane. This can be difficult for lipophilic compounds with poor solubility in water, thereby presenting a limiting step in the absorption process. The presence of lipids derived from food or lipid-based formulations in the intestinal lumen can influence the oral absorption of highly lipophilic drugs in many different ways. The solubility of the drug can be increased, due to the creation of various colloidal formations (vesicles, micelles). Drug solubilization can be influenced by lipid presence itself and by the simulation of physiological lipid processing pathways, leading to increased secretion of bile-salts and phospholipids [5,6]. The lipids can influence intestinal metabolism and influx/efflux transport. Studies showed that, in some cases, lipid excipients could improve drug absorption through the influence on the P-glycoprotein (P-gp) functioning [7]. Additionally, nuclear hormone receptors (NHR) were shown to play an important role in lipid trafficking and metabolism and, thus, in intestinal lipid and drug absorption, since they control a number of proteins (e.g., fatty-acid-binding proteins) that are involved in lipid/drug transport and metabolism [8,9,10]. Following oral administration, in many cases drugs pass through the hepatic vein on their way to the systemic blood, whereas highly lipophilic compounds may be transported through the intestinal lymphatic system. Lipids can also stimulate intestinal lymphatic drug transport, in which the drugs can bypass the first-pass hepatic metabolism and go directly to the systemic circulation [11]. After solubilization, drugs permeate through the intestinal membrane via passive diffusion/active transport through the enterocytes. For hydrophilic drugs with poor solubility in lipids, this step can be the rate-limiting in the absorption cascade, whereas, for lipophilic drugs, the unstirred water layer (UWL) in the proximity of the intestinal membrane is an obstacle for effective permeability. The diffusion of FA, monoglycerides (MG), and many other lipophilic molecules (including drugs and prodrugs) through UWL is significantly increased through micellar solubilization, prior to arriving to the enterocytes. It is likely these lipophilic molecules dissociate from the micelles prior to going into enterocytes or through binding to the transporter or vesicular-mediated transport of micelles. The following stage of drug absorption is leaving the enterocyte into the lamina propria, from where the drug is usually absorbed into the hepatic blood flow, unless it undertakes lymphatic transport, which is particularly important for lipophilic molecules, and it will be discussed in detail in part 2. The following section describes lipid processing pathways subsequent to oral ingestion. In addition to lipid processing pathways, this work provides an overview of lipid balance disruption in disease, pharmacological roles of certain lipids, and their role as lipid carriers in the drug delivery.

## 2. Lipid Processing Pathways

The digestion of exogenous lipids begins in the mouth via enzyme lingual lipase. It is followed by the gastric lipase in the stomach, combined with mechanical mixing. This is where PL and TG, alongside initial amphiphilic digestion products, such as FA, diglycerides (DG), and lysophospholipids (LPL), form crude emulsion [12,13]. Digestion continues in the small intestine, where pancreatic lipase, alongside its cofactor co-lipase, finalizes the disintegration of TG to DG, MG, and FA [14]. This enzyme mainly hydrolyses *sn*-1 and *sn*-3 positions of the TG, producing FA and MG [15]. PL digestion in the small intestine occurs via pancreatic phospholipase A_2_ (PLA_2_) enzyme, which hydrolyses the FA in the *sn*-2 position of the PL, producing a FA and LPL [16]. Exogenous lipids stimulate the secretion of biliary lipids, such as bile salts, PL, and unesterified cholesterol, which in turn stimulates the formation of vesicles and micelles that incorporate these exogenous lipids. This step allows for lipophilic drugs, as well as lipidic prodrugs, to incorporate into micelles, thereby increasing the solubilization of lipids/lipid-drug conjugates/lipophilic drugs. After the micelles break down, lipid digestion products become available for absorption to the enterocytes. Many molecules pass through the basolateral enterocyte membrane into the lamina propria on their way to hepatic blood flow. Others, such as lipophilic digestion products (FA, MG), are reassembled into TG/PL in the enterocyte, followed by incorporation to lipoproteins (rich with TG). Lipoproteins (LP), in this case, chylomicrons (CM) also then undergo exocytosis to the lamina propria, however they do not enter blood vessels, due to its large size that obstructs the passage through tight junctions between capillary endothelial cells; rather, LP enter leaky lymphatic capillary, lacteal [17]. The contents of lacteal are released to the systemic circulation via bigger lymph vessels, hence avoiding the hepatic blood flow. This intestinal lymphatic system allows the transport of absorbed lipids and other lipophilic molecules (drugs, liposoluble vitamins, prodrugs) from the small intestine to the blood circulation. The main advantages of designing a drug/prodrug that undergoes lymphatic transport is bypassing the portal blood flow and, thus, hepatic metabolism, and achieving lymphatic targeting in cases when its desired (e.g., treating cancer metastases).

**Figure 2:**
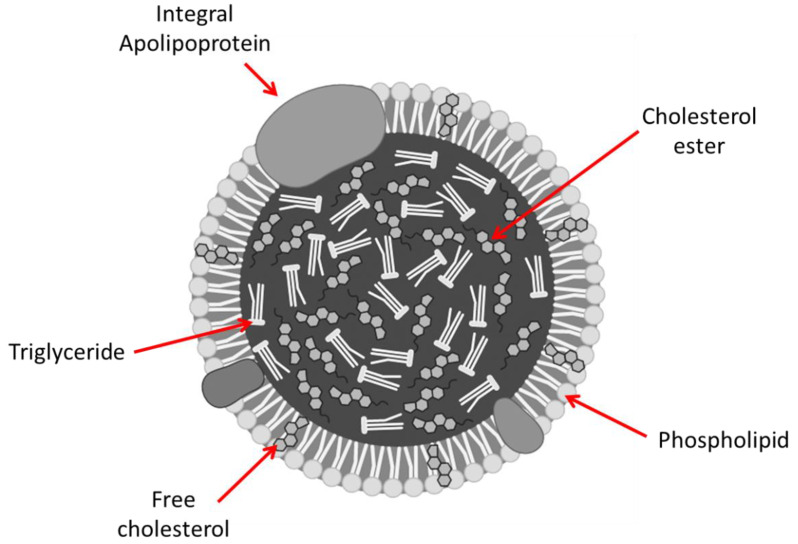
Lipoprotein structure.

Another important class of lipids is sterols, with cholesterol being the most familiar human sterol. Cholesterol is transferred from the intestine to the liver as a part of LP complex [18]. Figure 2 demonstrates the structure of LP. The role of LP is to transfer various lipids to the tissues [19]. According to its density, LP can be categorized by their density as ultralow density LP, chylomicrons (CM), very low-density lipoproteins (VLDL), low-density lipoproteins (LDL), and high-density lipoproteins (HDL) [20]. Another species of LP formed through the degradation of the VLDL and HDL are intermediate-density lipoproteins (IDL). CM is synthesized in the intestinal tissue and it is responsible for carrying the cholesterol and TG throughout the body. VLDL distribute the TG to various tissues. It is produced in the liver are transformed to LDL via lipoprotein lipase. LDL is responsible for getting cholesterol from the liver to other parts of the body; it contains apolipoprotein (apo B-100), which permits LDL binding to the LDL receptors on the cell surfaces. Lastly, the role of HDL is to carry cholesterol from the tissues back to the liver. Steroid hormones, mineralocorticoids, and glucocorticoids are synthesized from cholesterol, as well as bile salts. 

The misbalance in the presence of different lipid moieties and their oxidation products can be a sign of pathological condition. The role of lipids in pathological diseases and their therapeutic potential, as well as basic principles of lipidic prodrugs, are discussed in the following section. 

## 3. Lipid Role in Pathological Conditions and Their Therapeutic/Drug Delivery Use

**Figure 3:**
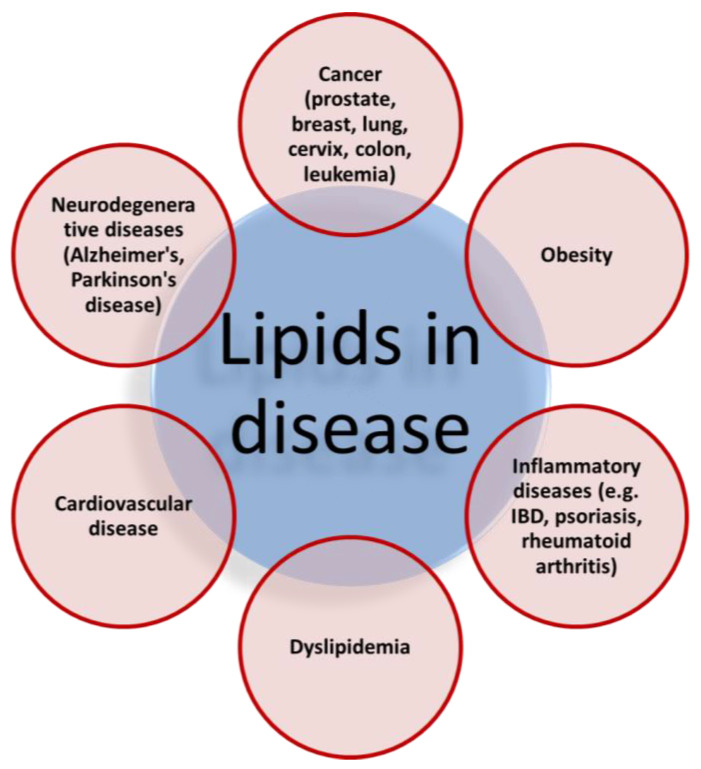
The representation of various diseases caused by disruption in the physiological lipid metabolism.

The application of lipidomics can help us to study lipid metabolism and it can help us to understand the underlying mechanisms of various metabolic diseases and the role of lipids in modulating homeostasis [21]. Figure 3 presents several diseases caused by disruption in lipid metabolism. The identification of specific lipid biomarkers and mechanism-based knowledge of particular metabolic lipid processing pathways in connection to different pathologies is basis for developing innovative therapeutic approach for treating different pathologies. In addition, we mention the use of lipid carriers in drug delivery, particularly the use of lipidic prodrugs. Lipidic prodrugs contain the drug covalently attached to the lipid carrier (e.g., FA, glyceride, PL, or steroid). This conjugation leads to higher lipophilicity when compared to the drug alone, and can thus lead to better pharmacokinetic profile and provide other significant benefits, such as improved absorption through biological barriers, extended blood half-life, selective distribution to tissues (e.g., brain, intestine), decreased liver first-pass metabolism, and overall improved bioavailability of the drug. Drug-lipid conjugates can also join the physiological lipid trafficking pathways and accomplish drug targeting to specific sites [22]. Exploiting the lymphatic transport is one of the mechanisms of lipidic prodrug incorporation to the lipid processing pathways, and it is highlighted in this paragraph.

### 3.1. Fatty Acids (FA)

FA contains hydrocarbon chains of various lengths and degrees of desaturation. Numerous lipids are synthesized from FA; for instance, PL and glycerides contain hydrophobic FA tail, as well as triacylglycerides, which are synthesized and stored in the state of elevated nutrient availability [23]. In the physiological conditions, de novo FA synthesis in humans originates in the liver, breasts during lactation, and adipose tissues [24]. However, in pathological conditions, such as cancer, it was demonstrated that FA biosynthesis plays an important role [25,26]. Fatty acid synthase was identified as the tumor antigen OA-519 in invasive breast cancer, in retinoblastoma [27], and it was also found in proliferating fetal tissues [24], signifying that the reactivation of cancer FA synthesis could mean a retreat to a less-differentiated embryonic state [23]. The elevated presence of FA in cancerous tissues could also be due to the increased metabolic demand in the cancer tissues or adaptation to decreased presence of serum lipids in the tumor environment [23].

The ratio of free FA turnover is reduced in the growing white adipose tissue. Adipose tissues and dietary saturated FA are in correlation with increased fat cell size and number. Adipose TG lipase inhibition is responsible for TG buildup, while the inhibition of hormone-sensitive lipase. the buildup of DG. Surplus triacylglycerols, sterols, and sterol esters are enclosed by the PL monolayer and create lipid droplets. The size and number of lipid droplet distribution is linked to obesity [28].

Unsaturated FA (UFA) are present in the human fat and they contain at least one unsaturated double bond. They could be monounsaturated (MUFA), if only one unsaturated double bond is present (oleic, elaidic acid), or polyunsaturated (PUFA), where a number of double bonds are present in the alkyl chain (i.e., ω-3 position: docosahexaenoic, eicosapentaenoic acid; or, ω-6 position: arachidonic, linoleic acid). PUFA are considered as essential FA, due to their significant physiological function, and due to the fact that they only come from exogenouse sourses (diet). UFA in general, have numerous physiological functions, such as maintaining functional biomembrane, promote cardiovascular wellbeing through lowering cholesterol/TG [29], anti-inflammatory effect, synthesis of prostaglandins/thromboxanes, and they promote brain functioning [30].

Omega-3 PUFA (4g a day) were shown to decrease the TG levels by 30%, respective of the baseline levels, and can thus be used in the treatment of pancreatitis [31]. However meta-analysis revealed that omega-3 PUFA supplementation did not decrease the risk of stroke, cardiac sudden death, myocardial infarction, and all-cause mortality [32].

Additionally, UFA were also found to have some endogenous anticancer properties, and some capability of promoting chemotherapeutic effect of anticancer drugs [33]. UFA have good biocompatibility, innate tumor-targeting features, and likely pharmacological activity in cancer treatment; therefore, they present good lipid carriers for prodrug development in cancer therapy. The conjugation of UFA with chemotherapeutic agent was shown to increase the lipophilicity of the drugs, which might enable the cellular uptake of anticancer drugs through passive transport (which is particularly important for water-soluble nucleoside drugs). Anti-cancer agent-UFA conjugates that entered clinical trials gemcitabine-elaidic acid (CP-4126), cytarabine-elaidic acid (CP-4055), and paclitaxel-DHA (PTXDHA) prodrug [33].

Lymphatic transport with FA is variable; whereas, short/medium-chain FA might be absorbed via hepatic blood, long-chain FA have a tendency to be reacylated into TG, integrated in CM, and transported through the lymphatics. FA-drug conjugates usually do not undergo lymphatic transport, due to extensive hydrolysis prior to absorption process, although some FA-drug conjugates have indeed shown successful lymphatic transport [34]. Testosterone is an androgen hormone that is subjected to high first-pass hepatic metabolism, which leads to very low systemic bioavailability following oral absorption; thus, testosterone by itself cannot treat male androgen deficiency syndrome. However when conjugated with an undecanoic acid, it showed an increase in systemic bioavailability up to 7% [35]. Testosterone-undecanoate undergoes lymphatic transport, thus avoiding the portal blood and first-pass metabolism (Table 1). In the blood, free testosterone is released from the prodrug and it is free to demonstrate its therapeutic effect [34,35,36].

### 3.2. Glycerides

Elevated plasma TG and TG-rich LP in non-fasting conditions have a significant role in various cardiovascular diseases, such as myocardial infarction, ischemic heart disease, and even death [37,38]. CM residues and LDL are entrapped by the arterial wall cells; in the general population, in people who do not have familial hyperlipoproteinemia, it was shown that atherogenesis can occur after the postprandial period [39]. High TG can be a marker for several types of atherogenic LP. TG-rich LP, such as IDL and VLDL, can be trapped within the arterial wall, while nascent CM and VLDL are too big to penetrate the wall [40]. It was suggested that lifelong high plasma TG-rich LP or their remnants are related to greater risk of ischemic heart disease, regardless of suboptimal HDL levels [41].

On the other hand, TG use in drug administration is important; TG can be used to enhance formulations of highly lipophilic drugs. They can be TG oils, various combinations of TG, DG, and MG. The sort of oil that is used in the formulation has significant impact on the formulation capacity to improve absorption [42]. Non-digestable lipids, such as sucrose polyesters, are not absorbed from the intestinal membrane, whereas digestable lipids, such as DG, TG, PL, FA, cholesterol, and some synthetic derivatives, are suitable components of drug formulation. They can be classified according to the degree of saturation, interaction with water, and the length of their carbon chain, which can be long-chain TG (LCT), medium-chain TG (MCT), as well as DG, MG, FA, PL, and others [42]. The effect of peanut oil (LCT), miglyol (MCT), and paraffin oil was studied on the absorption of probucol, hypocholesterolemic drug with high lipophilicity, with log P around 10 [43]. The highest bioavailability was obtained with LCT, peanut oil, then MCT, and lastly no absorption was evident for paraffin oil solution. However each drug formulation should be evaluated on a case-by-case basis.

Glycerides have an important role in the development of novel prodrugs with improved targeting features when compared to the parent drug. Oftentimes, the TG-drug conjugates undergo lymphatic transport and lymphatic drug delivery. In one instance, the immunosuppressant drug, mycophenolic acid (MPA) was conjugated to the sn-2 position of a glyceride [44]. This prodrug (2-MPA-TG) was designed to incorporate MPA to the TG deacylation–reacylation pathway, and increase the lymphatic transport of MPA following oral administration (Table 1). MPA lymphatic transport and systemic bioavailability was evaluated in dogs, following oral administration of MPA alone and MPA-prodrug [45].MPA levels in the lymph nodes and lymph residing lymphocytes were studied in order to determine the extent of MPA targeting to the sites of action within the lymph. The TG-MPA prodrug significantly increased lymphatic transport 288-fold, in comparison to the MPA; 36.4% of the dose was recovered in the lymph. The MPA level in the lymph nodes was increased 5- to 6-fold and in the lymph lymphocytes 21-fold *vs.* MPA administration alone. This study demonstrated that the TG-MPA prodrug could incorporate deacylation–reacylation pathway of the TG and successfully target the lymphatics through improved MPA uptake within lymph residing lymphocytes, resulting in the more effective immunomodulation of MPA [45].

### 3.3. Phospholipids (PL)

Lipids are strongly linked with amyloid precursor protein metabolism, resulting in amyloid-beta peptide (Aβ) formation, one of the key component of senile plaques, which characterize the pathological hallmark of Alzheimer disease [46]. Certain choline PLs were proposed as likely biomarkers of Alzheimer disease. It was shown that lysophosphatidylcholine, lyso-platelet, and choline plasmalogen activating factor levels during normal aging increase meaningfully; comparable but even more pronounced alterations were found in people with probable Alzheimer disease. Thus, higher choline-containing phospholipids in the plasma may be representative of a quicker aging process [47].

On the other hand, PL therapeutic effects were demonstrated in several diseases. It was shown in that certain PL moieties have therapeutic activity in ulcerative colitis [48,49]; some clinical trials exposed that the adding phosphatidylcholine (PC) to the colonic mucosa alleviates inflammatory activity [50]. One study, phase IIA, double blind, randomized, placebo controlled study including 60 patients with chronic active, non-steroid dependent, ulcerative colitis, showed that 6 g of retarded release phosphatidylcholine rich PL during three months period alleviates inflammatory activity that is caused by ulcerative colitis [50]. Another study showed that polyunsaturated PC is beneficial supplementary treatment for patient management in HBsAg negative chronic active hepatitis, where the condition is inefficiently controlled with standard doses of immunosuppressive therapy [51]. Dietary PC was also shown to alleviate the orotic acid-induced fatty liver in rats OA-, mostly through the reduction of TG synthesis in the liver and improvement of FA β-oxidation [52]. Dietary lecithin (mixture of phosphatidylcholine, phosphatidylethanolamine, phosphatidylinositol, phosphatidylserine, and phosphatidic acid) was shown to be protective in cholestatic liver disease in cholic acid-Fed Abcb4-Deficient Mice, through the drastic mitigation of the hepatic damage [53].

Oxidized PL are present in the inflamed tissues and they are taught to have an important role in the immune response modulation [54]. In most studies, oxidized PL have proinflammatory properties, however it was shown that particular PL oxidation products can display anti-inflammatory features. The derivatives of oxidized PL may, in fact, be a new treatment option for immune diseases (i.e., atherosclerosis, psoriasis, multiple sclerosis, and rheumatoid arthritis) [55].

Since PL have the innate ability to reduce inflammatory activity in certain disease, they make an interesting carrier for lipidic prodrug design. Our group investigated the PLA_2_-mediated activation of the PL-drug conjugate, and its potential use in inflammatory diseases, such as inflammatory bowel disease (IBD); in IBD intestinal tissues, the levels of PLA_2_ expression are elevated [56,57]. The aim of our work is to target the inflamed tissues with elevated PLA_2_ by oral PL-based prodrugs. Linking the drug directly to the sn-2 PL position showed the absence of PLA_2_-mediated activation [58]; however, once the linker was introduced the activation of the prodrug was possible [59,60,61]. Novel computational analysis was used to optimize the prodrug design (linker length) [62,63,64]. For instance, PL-indomethacin prodrug was orally administered to rats and the prodrug with 5-carbon linker (DP-155, Table 1) showed a 20-fold increase in free drug vs. the PL-indomethacin prodrug with the 2-carbon linker. Free drug was liberated in the intestinal lumen via PLA_2_-mediated hydrolysis [59]. Linker design is an essential parameter in the prodrug activation through PLA_2_. This was also demonstrated on an antiangiogenesis agent, fumagillin, by adding a 7-carbon acyl linker in the sn-2 position of the PL, the resulting PL-fumagilin prodrug was activated by local PLA_2_, and free fumagillin, free drug was released, demonstrating reduced angiogenesis in-vivo [65]. Through the smart design of PL-drug conjugates, we can enable the exploitation of endogenous PL processing pathway, through activation with the enzyme PLA_2_ [66].

### 3.4. Steroids

Cholesterol is the main sterol synthesized in humans. It can originate from the diet or can be synthesized *de novo* in the body [67]. As mentioned earlier, it has a variety of roles, from being a structural component of the cell membrane, part of different classes of LP, to being a precursor of steroid hormones, bile acids, and vitamin D [68,69]. Besides these physiological roles, cholesterol is found to play an essential role in the pathogenesis of some cancer types [70]; low cholesterol serum levels were found to be in connection with lung, cervix, breast, colon cancer, and leukemia, whereas high levels of cholesterol were associated with brain tumors [71]. For instance, prostate cancer is recognized as a lipid-rich tumor [72], with androgen receptor playing a main role in the development of this type of cancer. Genes for lipogenic enzymes can be regulated through androgen: the FA synthesis pathway and cholesterol pathway are highly influenced in this way, resulting in elevated synthesis of FA and cholesterol. Increased lipogenesis, as a result leads to elevated production of PL, cholesterol, and other cell membrane components, all characteristics of cancer cells. Additionally, bioactive lipids are also involved in prostate cancer progression [73]. Cholesterol is a precursor of estrogen, and elevated estrogen levels are linked with a greater risk of breast cancer [74]. In the growing cancerous cell, cholesterol is needed to make up cell membranes, which are synthesized faster in these tissues; the cholesterol is obtained from *de novo* sources or through LDL particles via high affinity receptor-mediated uptake. In many cases, cancer cells show high affinity to LDL particles vs. normal cells [74,75]. This increased demand for LDL by malignant cells and the overexpression of LDL receptors can be used for developing a novel targeted drug delivery system, among which are cholesterol-based prodrugs, as described in Section 4.

Cholesterol can be enzymatically transformed or oxygenated by free radicals to form oxysterols, 27-carbon derivatives of cholesterol [76]. They are connected to several diseases, e.g., atherosclerosis, Alzheimer’s disease, Parkinson’s disease, as well as cancer progression. Oxysterols also impact vascular ageing, by gathering in the of ageing blood vessel walls, they can stimulate monocytes and endothelial cells, and enable smooth muscle cells of blood vessels to proliferate, migrate, and act as fibroblast-like cells [77].

Atherosclerosis is a widely spread human pathology, associated with cholesterol and lipid metabolism [19]. The main risk factor for developing atherosclerosis is high cholesterol levels in the body, leading to deposits of fat in the arterial walls. Atherosclerosis therapy is dedicated to the reduction of cholesterol ester buildup. Prospective treatment to decrease cholesterol esters is targeted cholesterol ester hydrolase (CEH) delivery to hepatocytes using galactose-functionalized polyamidoamine dendrimer generation 5. The upward regulation of CEH prompts an increase in the cholesterol ester hydrolysis towards free cholesterol, which is consequently secreted as bile acids. This approach has been utilized for easing the accumulation of cholesterol esters in patients suffering from atherosclerosis [78].

Differences in the cholesterol uptake between normal and cancerous tissues could be used for developing cholesterol-based conjugates as a drug delivery system for targeting malignant diseases [79]. Cancer cells demonstrate an increase in LDL receptor activity, due to the high demand of cancerous tissue for cholesterol. This is due to fast growth of these cells or due to cell transformation mechanism. Hence, LDL is exploited as a carrier of anticancer agents, as a novel drug delivery approach for cancer therapy [79]. This method is based on the fact that, LDL is the endogenous transporter of cholesterol. Cholesterol in the body is mainly obtained through the LDL receptor-mediated endocytosis prevalently as cholesterol ester. Phosphotyrosine-cholesterol prodrug demonstrated efficacy against platinum-resistant ovarian cancer cells [80]. Ursodeoxycholic acid (UDCA) and zidovudine conjugation yielded a prodrug (UDCA−AZT), which was able to enter central nervous system, in comparison to free zidovudine (Table 1). Other advantages of this prodrug included enhanced antiviral activity, since it decreased zidovudine hydrolysis in the plasma, and improved the multi-drug resistance of zidovudine [81].

## 4. Discussion

Lipids are not only storage of body energy; they are fundamentally involved in the preservation and regulation of the cell function. Modifications in the lipid metabolism are linked with human diseases, such as cancer, diabetes, atherosclerosis, dyslipidemia, and neurodegenerative and infectious diseases. A number of drugs were developed to target specific lipid metabolic and signaling pathways, such as cyclooxygenase (COX) inhibitors or statins for the lowering of cholesterol. Researchers are pursuing particular regulators of lipid targets, such as nuclear hormone receptors (peroxisome proliferator-activated receptors, liver X receptor), phosphatidylinositol 3-kinases, ceramide kinases, and sphingosine [2]. An immunosuppressant drug targeting sphingosine-1-phosphate was approved for the treatment of multiple sclerosis, due to its targeting ability towards sphingosine-1-phosphate receptors, but not towards serine palmitoyl transferase, in contrast to its parent compound myriocin [83]. However, aside from glycolipids, very few antibodies are developed to recognize specific lipids; this is an important field that should be further investigated.

Lipids have vast potential in disease therapy, from being disease markers that enable drug targeted therapy, to being natively used as a supplement, as well as being part of the lipid drug formulation and used as a carrier for a particular drug. Some lipidic prodrugs (mainly FA-drug conjugates) were shown to be very successful in preclinical studies and, consequently, underwent clinical investigations. It was mentioned earlier that some UFA derivatives with chemotherapeutic agents entered clinical trials, most importantly gemcitabine-eladic acid [84], cytarabine-elaidic [85], paclitaxel-docosahexaenoic acid [86], and lipophilic docetaxel prodrug (MNK-010) [87]. In addition, cholesterol-siRNA prodrug (ARC-520) entered clinical trial for the treatment of chronic hepatitis B infection [88]. Commercially available lipidic prodrug product is TU for oral or intramuscular application in androgen deficiency [82]. A recent lipidic prodrug that reached the market is aripiprazole lauroxil and paliperidone palmitate an extended-release, long-acting intramuscular injection for the treatment of adults with schizophrenia [89,90]. Hence, lipidic prodrugs are a promising strategy for developing novel commercial products in the future [91]. Prospective direction for development of novel lipidic prodrugs containing glyceride/PL carriers could include even greater improved prodrug design, with linkers/spacers that would allow for controlled release of the parent drug from the prodrug complex [59,60,61,92].

Formulation lipids, such as LCT, MCT, as well as DG, MG, FA, and PL, are used to improve drug absorption. Their success in doing so is determined by several parameters, such as the amount of digestion products, digestion rate, and extent, as well as level of distribution of such products. The importance of combining lipidic prodrugs and lipid formulation design was recently demonstrated with lipidic prodrug, methotrexate-DG ester that was incorporated in the lipid bilayer of liposomes composed of egg (PC)/yeast phosphatidylinositol and demonstrated lower toxicity and slower lymphoma growth in mice when compared with methotrexate alone [93]. Lipids, both as carriers and formulation components (permeability enhancers), have a promising role as particularly diverse materials, with a vast space for discovery, development, and optimization in drug delivery.

## 5. Conclusions

Lipids may serve as biomarkers of diseases, as targets for drug molecules and as lipid carriers in the prodrug approach. Some lipids can be used as therapeutic supplementation to the drug therapy due to their innate pharmacological effect. All of this makes them valuable and diverse materials, with an encouraging role in future discovery, development, and optimization in drug delivery and therapeutics.

## Figures and Tables

**Table 1:** Molecular structures of lipid-based prodrugs.

Drug	Lipid-Drug Conjugate
**Testosterone [82]**	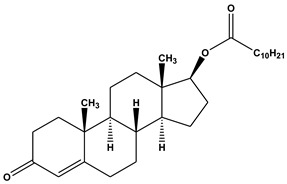 **Testosterone undecanoat**
**Mycophenolic acid (MPA) [45]**	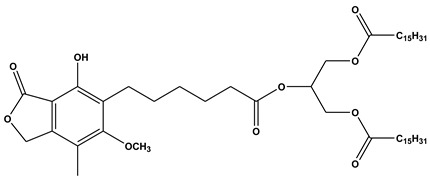 **2-MPA-TG**
**Indomethacin [59]**	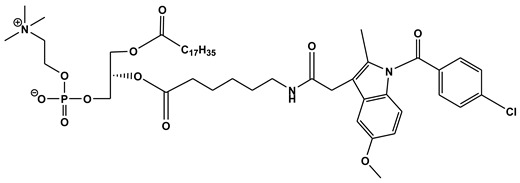 **DP-155**
**Zidovudine [81]**	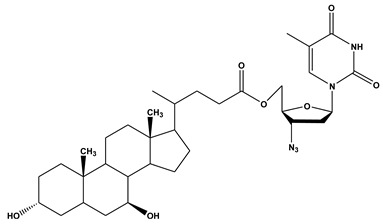 **UDCA−AZT**
